# A Web-Based Self-Management Intervention for Return-to-Work Among Persons With Common Mental Disorders on Sick Leave: Case Study of mWorks

**DOI:** 10.2196/92617

**Published:** 2026-07-13

**Authors:** Patrik Engdahl, Petra Svedberg, Ulrika Bejerholm

**Affiliations:** 1 Department of Health Science, Faculty of Medicine, Lund University, Sölvegatan 19, Lund, Skåne, Sweden, 46 735401126; 2 School of Health and Welfare, Halmstad University, Halmstad, Halland, Sweden; 3 ReLife-Centre of Mental Health and Recovery Across the Life Span, Lund University, Lund, Sweden

**Keywords:** mental health, mobile health, supported employment, vocational rehabilitation, primary health care

## Abstract

**Background:**

mWorks is a co-designed, web-based self-management intervention developed to empower persons with common mental disorders who are on sick leave during the return-to-work process. However, limited knowledge of how mWorks is delivered and engaged with in real-world settings constrains further development and implementation. In line with the Medical Research Council framework for complex intervention evaluation, such an approach is required to examine (1) contextual factors influencing implementation, (2) fidelity and variation in delivery, and (3) how service users and professionals experience and respond to the intervention.

**Objective:**

This study aimed to evaluate the process of implementing mWorks, specifically focusing on assessing the intervention’s delivery in relation to the context, implementation process, and mechanisms of impact.

**Methods:**

This single-case study was bounded by the delivery period of 10 weeks in a primary and specialist mental health service context. During this period, return-to-work professionals (n=2) and service users (n=6) collaborated to initiate mWorks usage. Both qualitative and quantitative methods were used to triangulate multiple data sources.

**Results:**

The pandemic and mental health problems posed contextual barriers, particularly during recruitment. However, perceptions of mWorks as a credible and relevant intervention facilitated its implementation. The delivery was performed according to plan, with minimal adaptations. All users adhered to the intervention, and dialogue meetings were highly valued. mWorks was used flexibly according to users’ needs, both during sick leave and at work. The potential impacts included a transformative process for users, fostering acceptance, self-esteem, self-compassion, and a sense of control. It also had the potential to prevent mental ill health, transform negatives into positives, facilitate disclosure of mental health, and support goal setting. The use of quantitative measures for empowerment, engagement, self-efficacy, depression stigma, and quality of life proved feasible and supported the assumptions and direction of results.

**Conclusions:**

The recruitment stage of the implementation program encountered significant contextual barriers. However, once the delivery stage began, the implementation of mWorks proved to be feasible. Despite the limited scope of this study, with its small number of participants, the triangulation of data suggests that both users and professionals benefited from mWorks.

## Introduction

Common mental disorders (CMDs), including depression and anxiety, are leading contributors to the global burden of health loss and are a major cause of work disability and sickness absence [1,2]. CMD-related sick leave represents a societal and economic challenge in terms of productivity losses and long-term exclusion from the labor market [3,4]. Despite a large body of evidence demonstrating that work participation is generally beneficial for mental health and well-being [5], there remains a persistent gap between the need for effective return-to-work (RTW) interventions and their availability and implementation in routine practice. However, emerging evidence indicates that digital RTW solutions show promise in reducing sickness absence days and alleviating depressive symptoms [6]. To address this gap, a digital RTW solution, mWorks, was developed that builds on a supported employment model adapted for CMDs, integrating motivational, cognitive, and time-use strategies, with a focus on strengthening empowerment and thereby improving RTW outcomes [7].

However, implementing an individualized and person-centered RTW model, such as supported employment, within a highly sectorized and disintegrated welfare system remains challenging. This system includes interacting actors such as primary and specialist mental health services (MHSs), Social Insurance Agency, Public Employment Service, and employers, which has proven challenging [8]. Within this complex organizational context, individuals with CMDs experience the RTW process and their interactions with RTW professionals from various organizations as unsupportive, as there is a lack of leadership and responsibility [9]. Additionally, RTW professionals and employers tend to prioritize the diagnosis, functional disability, and activity limitations rather than considering the individuals’ mental health, resources, and strengths in relation to their work [10-12]. Service users have voiced that this traditional kind of support diminishes their hope, sense of power, and belief in their work ability (self-efficacy), as well as their confidence in the ability of RTW professionals to assist them [13]. The observed deficits in hope and perceived control among users, together with the limited availability of person-centered models, provided the impetus for the development of mWorks and, more broadly, for the delivery of supported employment as a web-based self-management intervention.

Digital interventions have gained interest due to their potential to provide accessible welfare services regardless of geographical circumstances, time, and pace [14]. The transformation of cognitive behavioral therapy (CBT) into internet CBT has demonstrated success in reducing symptom severity [15] and is the most delivered mental health intervention online [16]. Furthermore, face-to-face CBT therapy and internet CBT with human support have shown comparable effectiveness for persons with mental health and somatic disorders [17]. Over the past decade, research within the RTW field has increasingly emphasized the need to digitalize RTW interventions [18-20]. However, the implementation of digital RTW solutions in clinical settings remains uncertain despite the potential of such solutions in MHSs. Considerable knowledge gaps exist regarding their optimal implementation in the targeted context, mechanisms of change, and value for users and RTW professionals. Implementation research has demonstrated that an unclear understanding of how novel interventions operate in a specific context often impedes the ability to embed such interventions into practice [21]. The RTW process for individuals with CMDs is complex and embedded in clinical and organizational contexts characterized by variability in resources, stakeholder involvement, and implementation conditions. Digital interventions delivered within such contexts may therefore influence both their implementation and uptake.

This case study investigated the implementation and use of the digital-supported employment intervention mWorks within routine MHSs. Given that RTW processes for individuals with CMDs involve complex interactions between multiple stakeholders, organizational structures, and contextual conditions, it was therefore selected, as it enables an in-depth examination of how mWorks is implemented and used in a real-world setting, including the contextual factors and underlying mechanisms that influence its integration and perceived value in practice [22].

## Methods

### Aim

The aim of this case study was to explore how the web-based self-management RTW intervention mWorks was implemented and used within routine clinical and organizational settings where RTW support is provided for persons on sick leave due to CMDs. Specifically, the study examines the following:

- What contextual factors influenced the implementation of the mWorks intervention and its mechanisms of impact?
- What was delivered in the mWorks intervention, and how was the delivery achieved in practice?
- How did service users and professionals respond to and experience interacting with the mWorks intervention?

### Study Design

This study resides in the feasibility stage of complex interventions according to the Medical Research Council (MRC) framework [23]. The study was bounded by a single case, which in this study regarded the implementation of mWorks delivery over a period of 10 weeks, using both qualitative and quantitative methods [24]. The 10-week period resembles the enabling phase of the previously mentioned supported employment model during which a relationship between the user and RTW professional is established, and when the engagement in the RTW process is initiated [18,25].

### Context and Setting

The case that constitutes the unit of analysis focuses on the delivery process of mWorks during a 10-week period [22]. RTW professionals and service users engage in 3 standardized dialogue meetings to facilitate their usage of the mWorks. The case was bounded by the context of the primary MHS organization in the south of Sweden, which has the authority to issue medical sick leave certificates.

Participants in this study include both RTW professionals and service users. The inclusion criteria for the RTW professionals were those working in primary MHS with regular RTW assignments of facilitating and coordinating the RTW process for service users within or connected to the primary care unit team. In Sweden, this position is typically known as rehabilitation coordinators (RCs) or employment specialists and can be held by occupational therapists, nurses, social workers, or psychologists. The County Council assigns the role of being an RC, and to become an employment specialist, one must provide supported employment. Purposeful sampling was used to recruit the RTW professionals. The first author (PE) and last author (UB) introduced information about mWorks and this study to managers of primary MHSs. We were also invited to inform the network of RCs 4 times during the study period of 2019 and 2021. UB also presented the study to the regional and national network of primary MHS and a national RTW network comprising practitioners, researchers, and service users. Notices were published in national papers and on social media in 2021.

### Recruitment

In 2019, 3 primary MHS centers entered the study. PE provided information meetings at the respective MHS unit, after which UB educated RTW professionals in January 2020 to start recruiting and delivering mWorks in February-March 2020, attempts that ended due to pandemic restrictions shortly thereafter. The primary MHS redirected its attention and resources elsewhere due to the ongoing COVID pandemic. However, in the fall of 2021, when social restrictions had eased, 2 units and 2 RTW professionals who remained interested in participation entered the study and were once again trained by PE. The inclusion criteria for service users to participate were being between the ages of 18 and 65 years and having a CMD, which includes depression episodes and recurrent depression disorder (F32-F32.2 and F33.0-F33.2), including depressive episodes inherent in bipolar disorder (F31.3 and F31.4) without psychosis, and/or anxiety disorders (F40-41) according to the *ICD-10* (*International Statistical Classification of Diseases, Tenth Revision*) code classification [26], being on sick leave (<2 years). The RTW professionals recruited service user participants and identified sick-listed individuals with CMDs who met the inclusion criteria. They obtained both oral and written informed consent from 7 service user participants, while 6 participants completed the intervention.

The RTW professionals (n=2) were employed as RCs, each working 20%‐25% of their time at 2 separate primary MHS units south of Sweden. They were 29 and 39 years old, respectively. Both professionals were women and held university degrees. The sociodemographic characteristics of the service users (n=6) revealed a mean age of 53 (SD 7.9) years, ranging from 44 to 64 years. All service users were identified as women. Among them, 4 were from Sweden, 1 from Germany, and 1 from Iraq. Moreover, 4 service users had a university degree, while the remaining 2 had completed upper secondary education. All service users had children, with 3 being married and 3 currently divorced. Additionally, 4 service users were on full-time sick leave, while 2 were on part-time sick leave. The mean duration of sick leave was 264 (SD 110) days, ranging from 80 to 365 days. All service users were sick-listed and recruited based on the inclusion criteria. Regarding their self-reported diagnoses, service user 1 reported anxiety and another diagnosis related to exhaustion. Users 2, 3, and 5 exclusively reported depression as their diagnosis. User 4 had depression and reported another diagnosis related to exhaustion. User 6 reported comorbidity of depression, bipolar disorder, and anxiety disorder. All participants completed the study and remained engaged through its full duration, with no attrition.

### The mWorks Intervention

The following intervention description is according to the TIDieR (Template for Intervention Description and Replication) checklist for describing clinical interventions [27]. The implementation program describes the preparatory and delivery stages of the mWorks intervention in the present case (Table 1).

**Table 1.** Description of mWorks implementation program.

Implementation step and components	Content	Time
Preparation		
Designing material for communication and delivery	Design of written and digital manuals for RTW^a^ professionals and service users Creation of mWorks website	5‐6 months prior to baseline
Initiating contact	Creation of mailing list Introduce mWorks broadly to stakeholder gathering Anchoring via telephone, email, and physical meetings	3‐4 months prior to baseline
Introducing concept	Introduces unit managers and RTW professionals to the project: introduce the mWorks concept with PPT via Zoom (Zoom Video Communications) and take part in written information about the project	1‐3 months prior to baseline
Mental health service unit responds yes or no to participation in the project	—^b^	—
RTW professionals initiate the recruitment of service users	Recruiting service users by purposeful sampling according to the inclusion criteria. Informing service users about mWorks and study participation with the aid of flyers and the website	1‐2 months prior to baseline
Education		
mWorks training during a half-day workshop with RTW professionals	Information about the evidence behind mWorks Presentation of the role of the staff Information regarding the delivery of mWorks Training of user administration and learning to navigate in mWorks	2‐4 weeks prior to baseline
Intervention start		
Dialogue meeting 1: introduction	RTW professionals deliver mWorks and the user manual Service-users use mWorks for 10 weeks	Baseline
Continuous guidance		
Dialogue meeting 2: follow-up	RTW professionals’ follow-up on service users	2 weeks
Continuous supervision	RTW professionals’ continuous support for service users Continuous supervision of implementation using memos (fidelity, dose, and reach) Supervision from the university to support RTW professionals regarding the implementation of the intervention	0‐10 weeks continuously
Follow-up activities		
Dialogue meeting 3: completion	RTW professional follow-up users’ experience of mWorks Follow-up dialogue with RTW professionals about their experiences of implementation, contextual differences, barriers and facilitators, and need for adaptation	2 weeks post intervention

a RTW: return-to-work.

b Not applicable.

**Figure 1.**
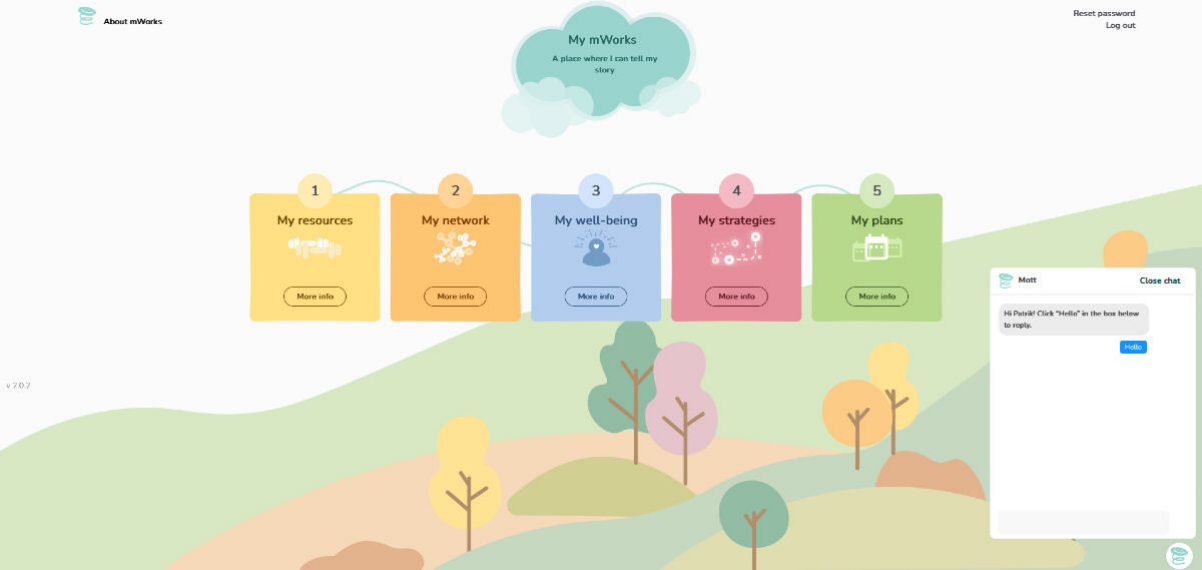
Overview of web-based self-management tool mWorks.

mWorks is a web-based self-management tool designed to support persons with CMDs with returning to work. It is compatible with smartphones, tablets, and computers. mWorks draws inspiration from supported employment and CBT according to the individual enabling and support model [28], and was coproduced with stakeholders described elsewhere [7]. During the first login session, a tutorial is presented to provide context and explain that mWorks is a support tool for RTW to be used at the individual’s own pace and preference. Additionally, an artificial intelligence (AI)–directed conversational agent named Mott provides bite-sized information through text dialogue to initiate usage. Upon entering mWorks after the tutorial (the original Swedish version of Figure 1 can be found in Multimedia Appendix 1), service users are nudged to start at “My mWorks” to facilitate a safe and pleasant digital space for transformation of their self-narrative relating to past and present experiences, exploring intrinsic drives using motivational interviewing. In order to create a positive user experience, mWorks uses positive language, with an absence of limitations, diagnoses, and medical orientation. Service users are free to navigate and use mWorks at their discretion, working through five steps: these steps are (1) My Resources helps service users identify their strengths and resources and compile a work profile that could be used in different occupational settings to effectively communicate their resources; (2) My Network makes it possible to map, gather, and clarify the meaning and function of important others and their contact information; (3) My Wellbeing helps to identify thoughts, emotions, and behaviors that affect their well-being at work, questions also support decision-making about disclosing their mental health to others, and psychoeducational digital film clips of fictional stories help to decrease personal stigma; (4) My Strategies identifies what difficult thought-emotion-behavior situations need to be addressed at work, an example list of work-health balance and cognitive strategies helps to support coping with these situations, and a list of preferred strategies to use is compiled; and (5) My Planning helps to pragmatically plan the RTW process, using goal-setting strategies, a to-do list, and a schedule to do so.

mWorks is a person-centered tool designed to foster ongoing self-management during the RTW process and at work, and its content features are updated as individuals progress. This study is limited to the delivery and engagement phase of the mWorks intervention. The role of RTW professionals consists of helping service users with usage and navigation within mWorks. Active delivery at the start of the intervention consisted of a minimum of 3 face-to-face meetings (Table 1) with RTW professionals in the MHS context assigned to deliver mWorks over 10 weeks. RTW professionals may freely navigate an administrative version but do not have access to the user’s self-management tool. Both professionals and users have their own manuals, while RTW actors, employers, and other involved parties may access concise but directed information available online, with or without a password.

The assumption is that mWorks may facilitate service users’ individual RTW process, enabling them to reflect on their own experiences and strengths and determine what is important to them during the sick leave process and when they work. This is expected to provide users with informed decisions, a view of their own beliefs and strengths, recognition of important others, strategies for well-being, and plans for sick leave and work. Thus, mWorks may increase user control during their RTW process, empower them, increase self-efficacy, improve their attitudes toward depression (stigma), reduce symptoms, and hopefully increase engagement in everyday life, quality of life, and global health. These are all factors that are likely to lead to a reduction in sick leave days.

### The Implementation Program

The implementation program describes the plan for implementing mWorks. The context in which sick leave certificates are issued in Sweden includes primary and specialist MHSs, as well as occupational health care. The implementation program focuses on preparatory planning, which is a critical part of the implementation process. This is followed by the education and delivery phases of implementation (Table 1).

### The Evaluation Plan

Evaluation guidelines according to MRC [29] and Saunders and colleagues’ [30] framework for process evaluation were used to organize the findings. A blueprint of our study can be viewed in Table 2. Both qualitative and quantitative data were collected during the 10-week delivery period, primarily through online questionnaires representing the constructs of interest, group, and individual interviews. Additionally, the mWorks web-based platform monitored the frequency and duration of usage. Service users’ and professionals’ responses and reflections about mWorks, as addressed in the My Memos questionnaire and interviews, provided a preliminary picture of the possible impact mWorks had on the participants.

**Table 2.** Blueprint of evaluation components.

Evaluation components	Research questions	Subquestions	Data sources
Context	What contextual factors influenced the implementation of the mWorks intervention and its mechanisms of impact?	What contextual barriers and facilitators affect the implementation of delivery?	Follow-up interviews, online questionnaire, documentation, and field notes
Implementation	What was delivered in the mWorks intervention, and how was the delivery achieved in practice?	Was mWorks delivered according to plan? What adaptations were made to fit the context? What dose did service users receive, and RTW^a^ professionals deliver?	Follow-up interviews, online questionnaire, documentation, and field notes Follow-up interviews, documentation, and field notes Online questionnaire and log data
Mechanism of impact	How did service users and professionals respond to and experience interacting with the mWorks intervention?	What were service users’ and professionals’ experience of mWorks? How does mWorks produce change and what are the tentative directions of the impact that mWorks may have on service users?	Follow-up interviews, online questionnaire, documentation, and field notes Follow-up interviews, online questionnaire, documentation, and field notes

a RTW: return-to-work.

RTW professionals were prompted to document the dialogue meetings throughout the study period. We provided RTW professionals with a document “My Memos,” which was developed by the last author (UB). My Memos has 8 items, with a mix of free-text answers and Likert scales (1-10). The question areas take inspiration from the evaluation framework of Saunders and the MRC and focus on fidelity, dose delivered and received, reach, and recruitment [30,31]. To mitigate recollection bias, RTW professionals were encouraged to respond to the questions in connection with each dialogue meeting and content feature that the service users used (Table 1). Finally, they were asked to summarize their answers at the end of the 10-week period.

RTW professionals and service users were interviewed at 10 weeks. The interview guides were based on the questions in My Memos. The follow-up interviews were semistructured and conducted to capture greater in-depth knowledge and understanding of the implementation [32]. The follow-up interviews were conducted by the first author (PE). Each follow-up interview was audio-recorded and lasted between 25 and 60 minutes. Field notes were conducted to capture additional information regarding context, the implementation process, and the mechanisms of impact [31].

Finally, service users were provided with a link to the online questionnaire (REDCap [Research Electronic Data Capture]; version 9.3.1) at baseline (T1) and at the end of the 10-week period (T2). The following psychometrically sound instruments that related to the assumptions made (see “The mWorks Intervention” section) were administered: Empowerment Scale [33], General Self-Efficacy Scale [34], Depression Stigma Scale [35], Montgomery-Åsberg Depression Self-Rating Scale [36], Generalized Anxiety Disorder [37], Profiles of Occupational Engagement Scale [38], and EuroQol 5-dimensions [39].

### Data Analysis

The first author (PE) transcribed the qualitative data verbatim, and the analysis was conducted by the first (PE) and last author (UB). The analysis procedure began with a thorough reading of the field notes, memos, and transcripts to gain an overall understanding of the data. Next, the different data sources, starting with the follow-up interview, field notes, and memos, were first organized using predefined evaluation constructs, after which coding within each construct proceeded inductively [31]. The different data sources were subsequently triangulated to compare similarities and differences between data sources, which helped in identifying common themes within the data corpus. For example, one service user explained how mWorks provided them with meaningful insights about themselves and was attributed to the mechanism of the change construct. Subsequently, content from the material that illustrated a similar pattern was coded to clarify nuances in the material according to inductive content analysis [40]. Codes with conceptual similarity were grouped into preliminary categories, which were iteratively compared and refined through discussion between the first and last author until broader themes were developed that captured recurrent patterns across the data. Coding differences were discussed in analytic meetings and resolved through consensus, with interpretations revisited against the original data material when needed. Reflexivity was supported through ongoing discussions between authors in which emerging interpretations and assumptions were critically examined, including consideration of how researcher perspectives might influence interpretation. The final step involved transforming the analysis into a coherent narrative representation. All authors critically scrutinized data analysis to mitigate interpretation bias and increase the trustworthiness and rigor.

Descriptive statistics were used to calculate the sociodemographic characteristics of service users and to explore the tentative direction of the impact mWorks may have had on users’ ratings between T1 and T2. Nonparametric and parametric paired-sample statistics were further used to corroborate the tentative direction of ratings, using the Wilcoxon signed-rank test and the paired-samples 2-tailed *t* test. The level of significance was set at *P*<.05, with a 95% CI. Data were analyzed using IBM SPSS version 28.0.

### Ethical Considerations

This study is part of a larger project with ethical approval from the ethics committee at Lund University, Sweden (application number 2017/324) and was performed in accordance with the ethical principles of the Helsinki Declaration of medical participants, including humans [41]. All study participants provided written informed consent before study enrollment.

## Results

### Contextual Factors

The primary contextual barriers included the COVID-19 pandemic, staffing limitations, and a lack of compatibility with the target group. Notably, the pandemic emerged as the most significant hurdle, severely affecting the recruitment of participants in MHS contexts. Primary MHS units had to make various adjustments due to the pandemic’s consequences. Their priorities shifted from general primary care toward preventing and treating patients with COVID-19 and administering vaccinations to the public. The social restrictions brought on by the pandemic meant that professionals rarely met with service users face-to-face to the same extent as prior to the pandemic.

Before, I had the patients here on site. Now, due to COVID, I cannot see patients anymore….You almost have to be some kind of telemarketer. You must have one, what to say, outreach activities, and you cannot really physically demonstrate what it (mWorks) is made of.

[RTW professional 1]

Another contextual factor regarded the characteristics and compatibility of the target group, which acts as an entry barrier. For instance, recruitment of the RTW professionals sometimes faced challenges in engaging presumptive service users as they were anticipated to be too exhausted, to lack interest, or to be low in energy to engage in new things. Professionals also mentioned that some service users would be reluctant to sit in front of a screen and make an effort when they get back home. In contrast, some participants were active and fully engaged and already had too many activities that limited their commitment. Additionally, one professional held the perception that mWorks might be perceived as a tool to push people toward RTW. Some service users similarly felt that mWorks had been created by society to push people back to work.

This (mWorks) presupposes that everyone wants to return to work quickly. Then you forget the cause of brain exhaustion. This is what I may have reacted to the most, it (mWorks) being too pushy.

[Service user 1]

Regarding contextual factors, such as staffing, the RTW professionals corroborated that their work assignment as RCs was tailored to coordinate and provide administrative support to the service users, making it a good fit for mWorks delivery. However, they sometimes found that introducing mWorks could result in questions being asked about mental health, well-being, and life in general, which they felt were not a part of their role or work assignment. Therefore, professionals suggested that mWorks could also be suitable for other professions within their units, such as occupational therapists or psychiatric staff on the team.

The support provided was helpful, especially parts that are probably best used with a therapeutic component….It would probably have been more helpful if I had the participant in therapeutic contact.

[RTW professional 2]

Furthermore, the RTW professionals worked 20% and 25% respectively of their full-time positions as RCs. One RTW professional suggested that, since they did not have therapeutic contact with presumptive participants, it impacted their ability to recruit more efficiently. Additionally, it was not entirely easy for professionals to grasp that mWorks was the user’s own tool. One professional felt that their administrative role in coordinating services made it difficult for them to respond to all reflections that mWorks elicited. However, they communicated with users to bring up concerns with other professionals at the unit. Initially, they also emphasized the importance of connecting mWorks to the Social Insurance Agency.

Wish it (mWorks) had a tab with information about sick leave, as users find it difficult and complicated. It would be great to have a module for sick leave similar to the rehab chain with days and other necessary information.

[RTW professional 2]

With regard to contextual perceptions of mWorks, participants generally described the intervention and its standardized delivery plan as acceptable and useful. Its design and content features were perceived to facilitate implementation, and the user interface was described as pedagogical, well-explained, and well-structured. One service user described mWorks as “pedagogical and visually appealing” to use. RTW professionals described mWorks as a useful tool to provide to users, which they felt could support users’ active engagement in their own rehabilitation and RTW process.

### Implementation Process

While COVID-19 did not restrict the initial preparation phase of creating delivery material, the pandemic was a severe implementation barrier for initiating contact and introduction of mWorks to MHS units (Table 1). The implementation was characterized by little involvement of one MHS team. Due to the pandemic, the information meeting was limited to a brief staff meeting during a 5-minute slot held by one RTW professional, which made the risk of forgetting it afterward very probable. Nevertheless, team engagement was perceived as valuable by both service users and RTW professionals, based on interview data from both groups. Once the MHS unit agreed to participate, the subsequent educational step went according to plan. Similarly, the active delivery and reception of mWorks functioned according to the program (Table 1), as documented in intervention delivery records by RTW professionals. Adaptations to the implementation concepts involved introducing additional contacts between dialogue meetings 1 and 2. Another adaption concerned involving other team professionals from the primary MHS, but neither were viewed to negatively impact service users’ engagement in mWorks and the RTW process, as reflected in RTW professional interviews.

The extent to which mWorks was perceived to be implemented in relation to program expectations was rated by professionals as 7 and 8 on a 10-point Likert scale. Implementation barriers, such as targeting the right group and not being able to meet up with presumptive users to introduce mWorks during the pandemic, limited ratings. Recruitment of service users was stated as challenging but to be expected in relation to the group of interest, with little energy and engagement, especially those with exhaustion symptoms.

Although RTW professionals were supported by introductions, support meetings, and an extensive manual, they did not have the resources to learn about the material by heart in order to deliver mWorks to service users with confidence. They simultaneously emphasized that it was not an intricate system and only required a little to become familiar with mWorks.

### Dose Delivered

The delivery of mWorks was possible to complete for all users. It was delivered in accordance with the delivery plan (eg, all dialogue meetings were conducted) and preserved users’ needs. RTW professionals and users perceived the dialogue meetings as ideal and valuable and that the time frame for delivery was adequate. Professionals rated the delivery as a 7 on a 10-point Likert scale in terms of alignment with the plan. The pandemic limited the feasibility of delivering the dialogue meetings, but it was not decisive for the delivery. On-site and face-to-face meetings were preferred over virtual meetings.

The RTW professionals delivered mWorks slightly differently. One professional noticed that some users were having trouble initiating usage of mWorks. Therefore, she preferred to have more frequent follow-up sessions prior to dialogue meeting 2. She adapted the delivery process in the initial stage by adding follow-up phone meetings to ensure that users had begun using mWorks.

In the beginning, I had more frequent follow-ups with the participant, which I experienced to be better. I had brief telephone follow-ups every week. But overall, I experienced that it worked well. Sometimes the patients have not started until the subsequent follow-up, and then it might be good to have closer contact in the beginning to just try make sure that they get going.

[RTW professional 1]

In accordance with delivery recommendations, support was adjusted to meet the needs, preferences, and interests of the service users. All users gave a rating of 10 out of 10 on the Likert scale for the quality of delivery by professionals. Despite this, users emphasized the importance of communication during delivery. They did not view mWorks as a quick fix, but rather as a tool that they could control and modify themselves, providing genuine assistance.

She has done it really well. I think it’s nice because she’s swamped, and still, she told me, “I will talk to Patrik”…and gave me an introduction when we started. We watched all the features, I cannot wish for anything better or more.

[Service user 1]

According to service users, the timing of delivery in relation to their sick leave status should be addressed individually. The users advised against delivery during the first few months of sick leave since cognitive exhaustion plays a key role.

No, but if I had access to it (mWorks) for the first three or four months, it would not have worked. I do not believe in that.

[Service user 5]

According to service users, the dialogue meetings with RTW professionals and all human support from other team professionals were critical for success. They emphasized that the combination of mWorks and human support is equally important.

These two complements, mWorks and psychiatric nurse. Unbeatable combination! I do not think you should choose one or the other, but you should combine them. A CBT therapist with this (mWorks), because then you would get the optimal fit.

[Service user 1]

The dosage of human support in relation to delivery was adequate for users getting started with mWorks. One professional mentioned that some users were positive about using mWorks without designated human support beyond dialogue meetings, while others were reluctant to end the contact.

### Dose Received

The overall usage of mWorks was diverse but aligned to individual users’ interests, needs, and preferences. All service users (n=6) logged into mWorks and participated in the dialogue meetings (approximately 5 hours). As monitored from the mWorks’ web-based system, the login frequency ranged from 4 to 20 during the study period, with a mean frequency of use of 9.8 (SD 6.18). The sum of logins during these 10 weeks was 49 for all service users (n=6). Notice, however, that the estimate is conservative since the monitoring was flawed for the first 2 users during the first couple of weeks. To complement the web data, interviewees also made an approximation of their login frequency, which was estimated to range from 8 to 40, and the frequency of minutes per login ranged from 7 to 30 minutes. In sum, usage in terms of frequency and duration varied among users during the engagement period of 10 weeks.

So it is not that I go to her (therapist) and get one dose and then another dose, but there is an active work going on (process). The space in between (meetings) is very useful to me.

[Service user 1]

Did not use it on a daily basis but repeated the steps in order for the content and what was elicited from and which knowledge got internalized. Because, yes, for it to get stuck in my head.

[Service user 5]

While one user used mWorks to engage in and moderate the entire RTW process, including sick leave and returning to work, another user started by getting to know the app during her 3 months of full-time sick leave but started to actively use mWorks when she returned to work. Yet another user focused on using all content features twice within a couple of weeks to internalize insights and strategies that were helpful for the parallel real-life RTW process. Service users rated their satisfaction with mWorks from 6 to 8. One user stated, “I have to say an 8, because when I have needed to, I have used what I needed” (Service user 3). RTW professionals confirmed users’ perceptions and estimated users’ satisfaction with mWorks at 7 on a 10-point Likert scale. The combination and consecutive order of content features provide a more accurate and clear view of factors contributing to their RTW process.

Similar to the overall usage, usage of different content features or steps varied significantly, as stated by both users and professionals. One user replied, “My Wellbeing, I used the most,*”* and mentioned how it helped break the vicious cycle of not prioritizing health and well-being during sick leave and at work. mWorks played a central role as a self-narrative for 2 of the users, “It was mostly about this with the story, to clarify this for oneself and others” (Service user 5). On the contrary, user 3 did not use My mWorks and stated no need to reflect on past and present experiences. She believed that addressing motivations was counterproductive since she was already motivated to RTW, “I don’t want to be there, I want to move forward” (Service user 3). She did not use My Wellbeing and stated, “I know exactly how I am...I do not need to concretize it further.” She preferred using My Network to increase her chances of asking for help from others, My Resources to cope with difficult situations at work, My Strategies to enhance work capacity, and My Planning to track progress. Furthermore, professionals rated My Strategies the highest (8 and 10) and My Plan the lowest (3 and 5) in terms of satisfaction ratings with the different features.

The patients talked a lot about this and then you could support and help them learn how to use these strategies at work.

[RTW professional 1]

One professional added that My mWorks, My Resources, and My Strategies were the most helpful features for her users. The consecutive order of, in particular, My mWorks and My Resources made a good start.

The dialogue meetings were also received differently. One service user benefited from each face-to-face meeting while another preferred one initial meeting and subsequently only some telephone support. Two of the 3 users interviewed (Service users 1 and 3) did not use the AI support Mott, as they did not find it challenging to navigate the tool independently.

### Reach

According to RTW professionals, among the users who participated, those who appeared to benefit most from mWorks were individuals who had already initiated their RTW process and perceived that work was within reach, as this was considered the group for whom mWorks was most suitable. One professional stated that “preferably those with a 25% range of sick leave status, but also at the 50% and 75%” (RTW professional 1). Furthermore, users reached were motivated to some extent and warranted a self-management digital solution. “I watched and read it (flyer), then I thought I absolutely wanted to do that. And so I got to” (Service user 1). Professionals stated that the reach was dependent on the MD’s diagnosis related to the sick leave. Persons with exhaustion who had difficulties managing their current life situation were considered less likely to engage in mWorks and evaluation activities. However, individuals with depression were believed to benefit the most from the program, as supported by user 3. This standpoint was corroborated by user 3, that persons with outright depression benefitted the most. Recruitment of the intended target group was addressed as challenging by all interviewees. It was considered a contradictory situation since persons with depression often experience insufficient capacity to take initiative and avoid situations.

So this whole thing to get into something (new). For me personally, it just came last on the list, so even if I knew it (mWorks) might help me, I did not have the ability and motivation, “I don’t care,” that you only exist. In the beginning, you just try to deal with your anxiety and depression, like everything else just comes last on the list.

[Service user 3]

One user emphasized that mWorks needs to be described as “not something huge” at the recruitment stage, that it is not obligatory but builds on user preferences, control and strengths.

### Mechanisms of Impact

The reflections and responses of service users regarding the impact of mWorks are presented below (Textbox 1). In general, mWorks benefitted users with their own private space to turn to for reflection and documentation related to life events, sick leave, and work. Similarly, users felt relieved that mWorks was available regardless of time and place. The opportunity for self-reflection and overview of the RTW process enabled users to gain authentic insights about themselves and identify unhealthy thinking and behavioral patterns. They also recognized the importance of their well-being before sick leave and learned new strategies to prevent mental ill health in the future.

The story that has reached closure, and at the same time a continuation, about how you, that with strategy (My Strategies), and what one should attend to. Not to revisit certain behaviours that are not that helpful, for example, or ways of thinking.

[Service user 5]

Textbox 1. **Content themes related to mechanisms of impact.**

- Provides with own private space to turn to for reflection and documentation
- Reveals authentic insights about oneself and their residence concerning the entire return-to-work process
- Prevents mental ill health from unfolding
- Turns negatives into positives
- Makes it possible to explain circumstances (mental health and return-to-work) to others
- Supports goals and following them through
- Generates user control and agency

Initially, users did not see any positives about themselves and RTW, but mWorks helped them turn negatives into positives. It also allowed them to recognize their support network and explain their circumstances to others who did not understand their predicament.

At the same time, I also think it (mWorks) was helpful in how I explain it to others who do not understand, or who do not know. So those were the two things that I thought were great.

[Service user 3]

In addition, mWorks could be an essential platform for supporting goals and following them through as it becomes more likely to pursue their commitments. In sum, service users explained that the accrued insight produced beliefs about themselves which generated experiences of having “self-esteem,” “acceptance,” and “self-compassion.” By virtue, RTW professionals perceived that mWorks generated user control and agency, which users corroborated.

mWorks has helped me to persist and understand the importance of healing throughout the process. Perhaps it was these questions that pushed me a little further, even though I haven’t reached that point yet. Maybe they have strengthened me? “Yes, that sounds really nice, but not right now....” This has boosted my self-esteem because I have come to accept that I can play a role and believe in my abilities.

[Service user 1]

RTW professionals’ attitude toward mWorks was generally positive since it targeted the entire RTW process and that they could offer service users the opportunity to work with a support tool that could be used on their own and in between sessions.

I thought it was nice that I would get some kind of tool to be able to give patients to work with as homework almost. And I thought it would be fun to start using mWorks.

[RTW professional 1]

**Figure 2.**
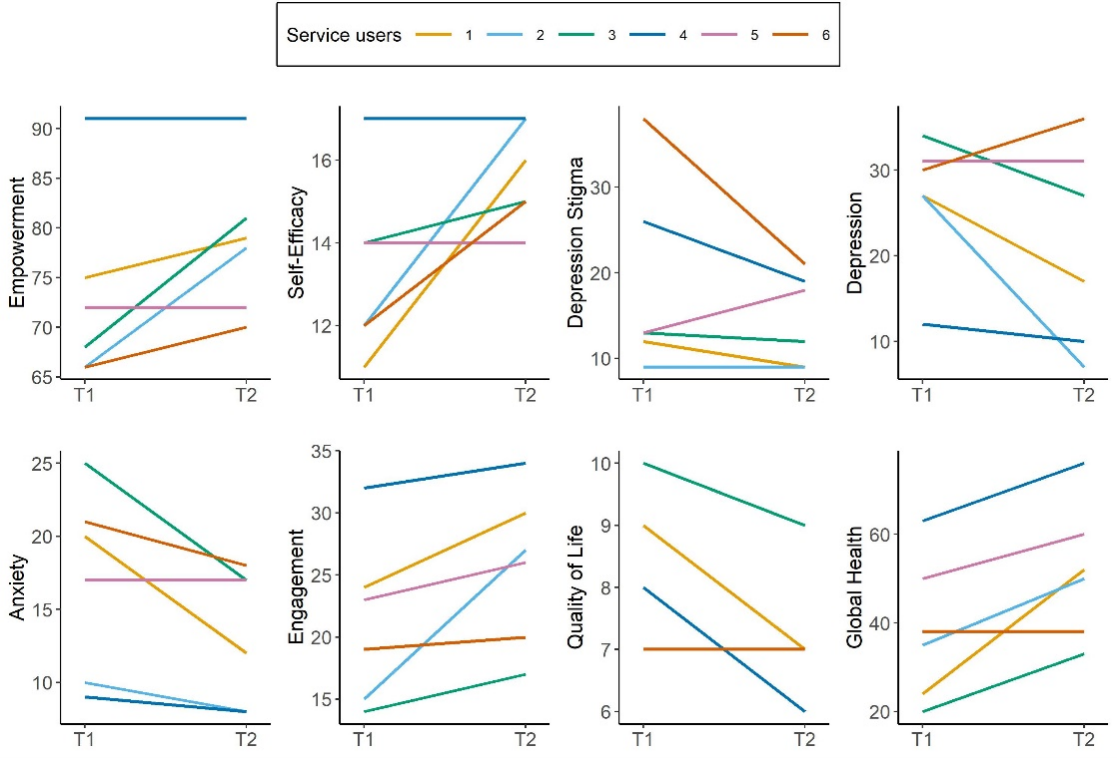
Service users’ individual trajectories of sum scores between T1 and T2 in relation to different measurements using spaghetti plots (low quality of life scores indicate higher quality of life).

The results related to mechanisms of impact indicated that the procedural feasibility of using questionnaires was acceptable, as all service users (n=6) completed the survey at both T1 and T2. The tentative direction of results according to users’ experiences rated in the questionnaires further validated the sensitivity of constructs chosen and helps to understand possible mechanisms of impact and outcome of mWorks. The individual trajectories and paired sample statistics support the initial assumptions that the mWorks intervention can enhance service users’ empowerment, self-efficacy, and attitudes toward depression (stigma). This is demonstrated by the positive changes in measurement scores observed between T1 and T2 (Figure 2). Notably, the majority of users scored lower in their levels of depression and anxiety at T2. In terms of health-related quality of life, most users showed a positive trajectory, apart from one who reported the same score at T2 (Table 3). This trend was also evident in global health measures. The positive trends observed in the individual trajectories were further supported by the paired sample statistics (Table 4). Importantly, the sensitivity analysis of empowerment highlighted that the subscale rating of optimism and control changed significantly (*Z*=–2.032, *P*=.04; *t*_5_=–2.739, *P*=.04).

**Table 3.** Descriptive statistics of service users’ (n=6) self-ratings of measurements at baseline (T1) and 10-week follow-up (T2).

Construct	T1		T2	
	Score, mean (SD)	Score, median (IQR)	Score, mean (SD)	Score, median (IQR)
Empowerment	73 (9.51)	70 (66‐91)	78 (7.45)	78 (70‐91)
Self-efficacy	13 (2.16)	13 (11‐17)	16 (1.21)	16 (14‐17)
Depression stigma (personal)	18 (11.22)	13 (9‐38)	15 (5.32)	15 (9‐21)
Anxiety	17 (6.36)	18 (9‐25)	13 (4.63)	14 (8‐18)
Depression	27 (7.73)	28 (12‐34)	21 (11.78)	22 (7‐36)
Engagement in everyday life	21 (6.68)	21 (14‐32)	26 (6.3)	26 (17‐34)
Quality of life	8 (1.17)	8 (7-10)	7 (1.09)	7 (6-9)
Global health	38 (16.11)	36 (20‐63)	51 (15.49)	51 (33‐76)

**Table 4.** Nonparametric and parametric paired-sample statistics (n=6).

Construct	Wilcoxon 2-sample paired signed rank test		Paired sample *t* test.	
	Z	*P* value	*t* test (*df*)	*P* value
Empowerment	−1.841	.06	−2.356 (5)	.65
Self-efficacy	−1.841	.07	−2.445 (5)	.06
Depression stigma (personal)	−1.214	.23	−1.244 (5)	.27
Anxiety	−2.032	.42	2.564 (5)	.05
Depression	−1.483	.14	1.407 (5)	.22
Engagement in everyday life	−2.207	.03	−2.730 (5)	.04
Quality of life	−1.890	.06	2.907 (5)	.03
Global health	−2.032	.04	−3.575 (5)	.02

## Discussion

### Principal Findings

This case study demonstrated that it is feasible to deliver mWorks as a web-based self-management intervention to service users with CMDs on sick leave within a primary MHS context. Overall, qualitative and quantitative findings suggest potential benefits in terms of user engagement, empowerment, self-efficacy, and quality of life during the RTW process. Once initial contextual barriers related to the recruitment of MHS units and participants were overcome, mWorks proved to be a valuable tool for RTW professionals to offer service users, complementing person-centered practice. However, given the small sample size and feasibility design, these findings should be interpreted cautiously and are intended to inform further research rather than indicate effectiveness.

Initial implementation was challenged by substantial contextual barriers, particularly related to recruitment of both service users and participation, exacerbated by the COVID-19 pandemic. The recruitment of contexts was severely hindered by the pandemic. This may indicate hesitancy among service users regarding acceptance. However, similar recruitment challenges have also been reported in studies of digital and RTW interventions, reflecting limited readiness and competing organizational priorities [42]. Once these barriers were partially addressed through engagement with County Councils in the implementation steering group and the development of a mWorks website to further promote the project, interest in mWorks emerged across multiple stakeholder groups, although willingness to participate in research remained limited. These findings suggest that successful implementation requires early and broad organizational engagement, including full team involvement within primary MHSs.

Timing of delivery and level of professional support appeared critical for facilitating engagement with mWorks. Findings suggest that later initiation during sick leave and flexible, user-centered support may improve uptake, particularly among individuals with exhaustion-related symptoms. This aligns with prior research emphasizing that symptom severity and fluctuation in mood that result in lack of energy and time have been revealed as an essential barrier to engagement with digital support tools to decrease depression in the workplace [43]. Therefore, providing well-timed and flexible support based on service users’ needs has been emphasized as a critical factor in facilitating usage [42,44]. Although exhaustion was not initially considered within the target group for mWorks, the current findings indicate that service users benefited from mWorks and were able to use it at their own pace and according to their preferences. As such, mWorks may be better suited as an ongoing, flexible support tool rather than a time-limited intervention. In addition, future education to RTW professionals should highlight critical ingredients for recruitment and delivery to ease learning among professionals with limited resources. Evaluation of web-based intervention for RTW suggested that professionals within the MHS lacked motivation to work with digital solutions [45], which might reflect the hesitancy for enrollment. Thus, it is essential to further understand the barriers and facilitators to engage and recruit professionals and organizations for web-based interventions.

As suggested in previous research, human support is essential for online delivery [46]. During the pandemic, face-to-face dialogue meetings were in some cases replaced with telephone sessions, which professionals perceived as less beneficial. However, this adaptation did not appear to affect service users’ satisfaction, as interviewees rated delivery quality at the highest level. Earlier formative research on mWorks [20,47] highlights the importance of identifying optimal formats for providing human support in digital interventions, a topic widely discussed in relation to service user engagement [48]. RTW professionals have been suggested to overvalue face-to-face meetings, whereas digital or telephone formats may be sufficient [47]. Similarly, trials and meta-analyses indicate that telephone-delivered CBT is comparably efficacious and acceptable to face-to-face CBT [17]. This study supports the value of human support, although the acceptability of telephone-based delivery remains uncertain.

Finally, findings suggest that mWorks may support empowerment-related mechanisms, particularly increased optimism and perceived control. This provides tentative support for the causal assumption outlined in this study and prior formative work [20]. These changes may reflect structured reflection on goals, strengths, and RTW strategies, facilitating self-management and greater perceived control over the RTW process. This is consistent with evidence on the importance of locus of control in digital mental health interventions [49] and prior work linking empowerment to RTW outcomes [50,51]. However, the findings remain preliminary and should be interpreted cautiously. This aligns with prior theoretically promising RTW interventions, including the Integrated Health Care and Vocational Rehabilitation trial, which, despite earlier supporting work, found no improvement in RTW outcomes compared with usual care [52]. Overall, this suggests that empowerment processes may not readily translate into sustained RTW effects and require further study in larger trials.

### Methodological Considerations

Adhering to the updated MRC guidance supported transferability through consideration of context, implementation processes, and mechanisms relevant to applicability in other settings. It also facilitated comparability through established evaluation constructs commonly used in complex intervention research and was therefore particularly well-suited due to its alignment with the study aims [23,31]. The credibility of the findings was strengthened through triangulation, involving multiple participant groups, datasets, and researchers. However, it is important to note that despite the positive results indicated in this study, causal inferences cannot be made due to the small-scale nature of the study and the limited sample size. Furthermore, the sample group primarily consists of middle-aged women, which is in harmony with the target group of persons on sick leave due to CMDs and may strengthen the overall transferability of our findings. Nevertheless, it is crucial to also include the younger population in the sample. Rigorous large-scale evaluations are necessary to assess its effect on outcomes and the underlying mechanism of change. Furthermore, this study was bounded by a 10-week timeframe to evaluate the initial delivery process of mWorks. Therefore, the insights regarding mWorks should be considered within the context of this limited timeframe. It is vital to evaluate the long-term usage of mWorks to gain a more comprehensive understanding.

### Conclusion

When the contextual implementation barriers related to the COVID-19 pandemic and recruitment were overcome, the implementation and delivery of mWorks were found to be feasible to implement and deliver in a primary MHS context. Both service users and professionals reported benefits from mWorks, as service users experienced increased engagement during the RTW process. Clinical and research implications for future implementation should focus on the initial stages of recruitment and involve the entire team during the education phase. The case study provides preliminary indications of potential mechanisms of change associated with mWorks in relation to the RTW process, particularly engagement and empowerment processes, with possible implications for self-efficacy, mental health, and quality of life. However, given the small sample size and feasibility design, these findings should be interpreted cautiously and do not allow for conclusions about effectiveness. Further research is needed to fully understand the process of implementing mWorks in other contexts and to establish the efficacy of the intervention.

## Supplementary material

10.2196/92617 Multimedia Appendix 1 Overview of web-based self-management tool mWorks (original Swedish).

## Abbreviations

AI: artificial intelligence
CBT: cognitive behavioral therapy
CMD: common mental disorder
*ICD-10*: *International Statistical Classification of Diseases, Tenth Revision*
MHS: mental health service
MRC: Medical Research Council
RC: rehabilitation coordinator
REDCap: Research Electronic Data Capture
RTW: return-to-work
TIDieR: Template for Intervention Description and Replication
