# Supplementary figures and images for: A Web-Based Self-Management Intervention for Return-to-Work Among Persons With Common Mental Disorders on Sick Leave: Case Study of mWorks
Source: JMIR Form Res. 2026 Jul 13;10:e92617. doi: 10.2196/92617 (PMC13362875; doi:10.2196/92617)

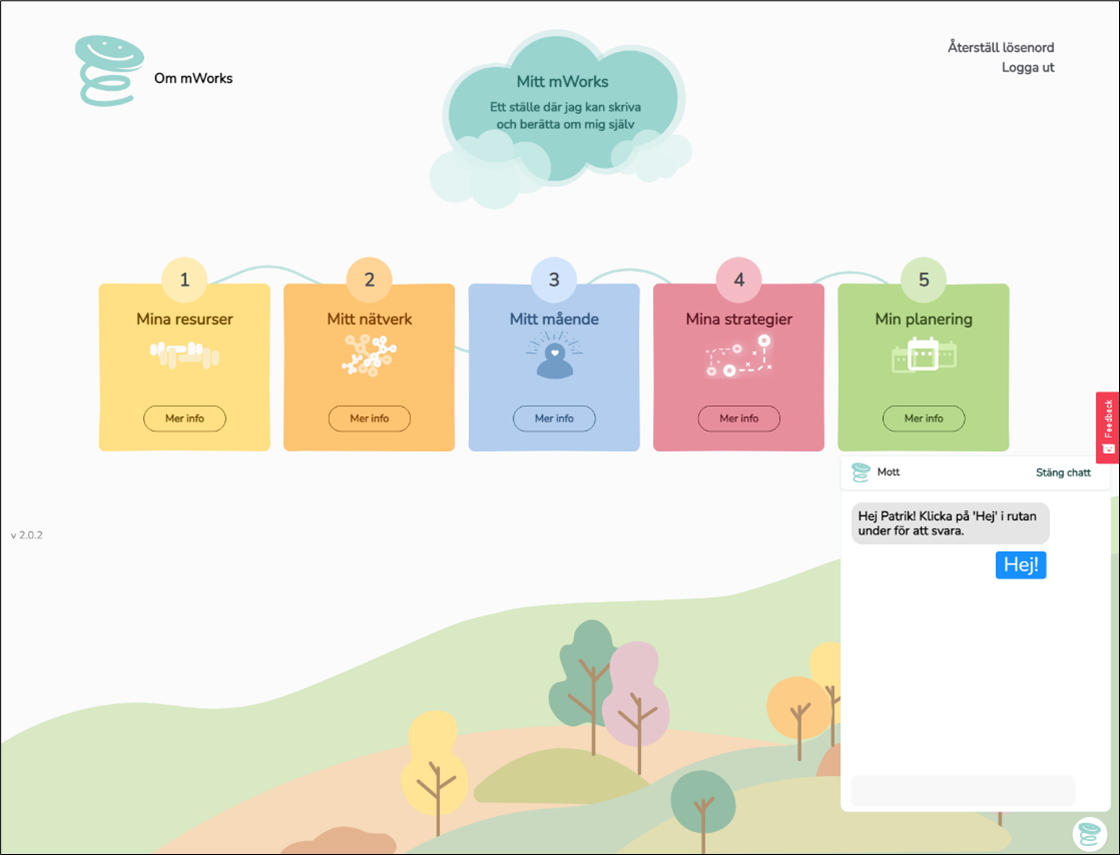

Supplement: Multimedia Appendix 1 [file formative-v10-e92617-s001.png]
